# Knowledge and practices of South African oral

**DOI:** 10.4102/hsag.v29i0.2632

**Published:** 2024-08-08

**Authors:** Zara Chothia, Ntombizodwa R. Nkambule, Ahmed Bhayat, Mpho Morule

**Affiliations:** 1 Department of Community Dentistry, Faculty of Health Sciences, University of Pretoria, Pretoria, South Africa

**Keywords:** dentistry, health education, dental, COVID-19, COVID-19 vaccines, dental staff

## Abstract

**Background:**

Severe Acute Respiratory Syndrome Coronavirus 2 is a recently discovered virus responsible for causing coronavirus disease 19 (COVID-19). No study has been carried out on South African oral healthcare workers (OHCWs) regarding their knowledge and practices with regard to COVID-19 and its vaccine.

**Aim:**

This study aimed to determine the knowledge, attitudes and practices of South African OHCWs regarding COVID-19 and its vaccine.

**Method:**

This was a cross-sectional study, which utilised an online questionnaire. The link to the questionnaire was sent via email and social media platforms. There was a total of 8056 OHCWs, and a minimum sample size of 367 was required. All information was confidential and anonymous.

**Results:**

A total of 327 OHCWs participated with a mean age of 43 years (±12.23) and the majority (60%) being general dentists. Less than half (42%) had obtained additional postgraduate qualifications while 57% were employed in the private and 24% in the academic sectors. Almost two-thirds (60%) obtained a ‘good’ knowledge score. Overall, OHCWs displayed positive attitudes towards COVID-19 and the majority implemented appropriate infection control protocols at their place of work. The majority (87%) reported to be vaccinated and of those who did not vaccinate, 34% cited concerns about possible side effects as a reason for not vaccinating.

**Conclusion:**

Respondents displayed gaps in their knowledge. There was a positive attitude towards the prevention of COVID-19, and almost all participants reported to have implemented the necessary infection control methods.

**Contributions:**

This study’s contribution to research was to identify gaps in the knowledge and practices of OHCWs with regards to COVID 19 and its vaccine. Once these gaps have been identified, measures will be put in place to address them.

## Introduction

The discovery of severe acute respiratory syndrome coronavirus 2 (SARS-CoV-2), the virus that causes coronavirus disease 19 (COVID-19), officially occurred in January 2020 (Li et al. 2020). Since then, it has spread across the globe and is considered a worldwide pandemic. As SARS-CoV-2 has been recently discovered, there are many uncertainties regarding the knowledge and attitudes of oral healthcare workers (OHCWs) regarding this virus.

Researchers managed to gain significant knowledge on the genetic code for the virus, which led to the development of the COVID-19 vaccine, and in 2020, the first fully approved vaccine authorised for emergency use was rolled out (Kew & Sguazzin 2022).

As a result of the nature of the spread of this virus, OHCWs were considered high-risk for the spread of this disease. Therefore, it was essential to determine the knowledge of OHCWs regarding the transmission, spread and prevention of the virus in their daily practices as well as their attitudes and practices around the virus and its recently discovered vaccines.

It is documented that healthcare workers (HCWs) who have a lack of confidence, disinclination, or an unfavourable attitude towards the vaccine will transmit attitudes characterised by argument or controversy and will tend to infrequently recommend vaccination (Kabamba et al. 2020). It is understandable that there are unanswered questions regarding the virus and its vaccine and there are gaps, which need to be filled as deeper scientific detail may be beyond us without further studies.

There has been a roll-out of vaccines globally and as of March 2022, 11.2 billion vaccines have been administered worldwide (Ritchie et al. 2020). South Africa (SA) was one of the first countries in Africa to receive the vaccines with the first batch administered in February 2021. The three vaccines secured by SA were the AstraZeneca (Oxford), Johnson and Johnson and Pfizer (BioNTech) COVID-19 vaccines.

It is necessary to gauge OHCWs’ perceptions of this vaccine and possible side effects in order to create awareness and reasons for their willingness or reluctance towards the vaccine.

Thus, this study sought to evaluate the levels of knowledge, attitudes and practices regarding COVID-19 and its vaccines among South African OHCWs.

The impact of COVID-19 on dentistry, both long-term and short-term, has not yet been completely understood. This study will provide baseline information regarding the knowledge, attitudes and practices of OHCWs towards COVID-19 and its vaccines.

## Materials and methods

This was a cross-sectional analytical study, which utilised an online questionnaire. A total of 8056 oral healthcare professionals registered with the HPCSA in 2021 were invited to participate in this study. An OHCW was defined as personnel who were involved in providing direct face-to-face delivery of dental treatment to patients. It included dental specialists, general dentists, dental therapists, dental hygienists as well as dental assistants. It included OHCWs who were in private practice, the public sector, the military sector and those employed in the dental teaching hospitals in SA.

The questionnaire was anonymous and included questions from the existing questionnaires, which was adapted from previous studies (Khader et al. 2020; Limbu, Piryani & Sunny 2020; Mustafa, Alshali & Bukhary 2020). It comprised of four sections; section A (questions 1–8) was on demographics and included the age, year of qualification and place of practice among others. The year of qualification was calculated by subtracting the year they graduated from 2022, which was the year in which the study was conducted.

Section B (questions 9–21) was on the knowledge of COVID-19 and its vaccine. This comprised 14 multiple-choice and true or false questions on general knowledge about COVID-19. Some of these questions had two correct answers and as a result, there were 18 correct answers. In the questions with two correct answers, OHCWs were scored two points for marking both correct options and one point for marking one correctly. ‘Poor’ knowledge was defined as a score of zero to seven, ‘average’ as eight to 11 and ‘excellent’, more than 12. The possible range was from 0 to 18.

The knowledge of the vaccines consisted of four questions. Each question had one correct answer; hence, the minimum score was zero and the maximum was four. The total knowledge score was calculated by combining the COVID-19 knowledge score to the vaccine score. The minimum total score was zero and the maximum was 22. These scores were categorised as ‘poor’, ‘average’ and ‘good’. ‘Poor’ was defined as a score of seven and below, ‘average’ between eight and 15 and ‘good’, 16 and above. Section C (questions 22–38) assessed the attitudes towards COVID-19 and its vaccine and section D (questions 39–48) recorded the practices regarding COVID-19.

Various methods were used to distribute the questionnaire to OHCWs. These included the Med-Bay platform, a database of all registered HCWs, WhatsApp groups, congresses and conferences and through snowball sampling. To ensure that there were no duplicate responses, participants were asked to only complete the questionnaire once and if they had received it from any other source, they were asked to decline its acceptance.

Based on the number of OHCWs registered in SA, with the confidence interval of 95%, using the Raosoft sample size calculator, the minimum sample size required was 367. The study population was further stratified into different cadres. Data were collected using a convenience sampling and a snowball technique between June and July 2022 via the Qualtrics data capturing system. All participants were informed that their personal information would not be saved on any server that could be accessed through the internet and all personal data would be saved on a password-protected hard drive locked in a filing cabinet, which required a key or an access code, thereby ensuring security and no sharing of personal information.

Each questionnaire was assigned a unique serial number. Once data were collected on the Qualtrics platform, they were exported to Statistical Package for the Social Sciences (SPSS) version 28 for analysis. Quantitative variables were summarised as proportions, frequencies, and means with their standard deviations, ranges and percentages. The chi-square test was used to evaluate the association between variables. Analysis of variance (ANOVA) and Kruskal–Wallis tests were used to compare the continuous data and mean age score. The level of significance was set at *p* < 0.05.

### Ethical considerations

Ethical clearance to conduct the study were obtained from the University of Pretoria, Research Ethics Committee of the Faculty of Health Sciences, ethics reference number 699/2021.

## Results

A minimum of 367 OHCWs were required and after 2 months of questionnaire submission and reminders, a total of 327 participants completed the questionnaire. The mean age was 43 years (±12.23; 20–76) and 69% received their latest qualification between 2005 and 2021. The majority (60%) of participants were general dentists, 16% oral hygienists, 11% dental assistants, 9% dental specialists and 4% dental therapists. More than two-thirds (69%) of respondents had obtained postgraduate qualifications and of these, 55% (75) had obtained a postgraduate diploma. The sample size for each question varied as not all respondents answered all the questions. The majority (57%) of respondents were employed in the private sector, 24% in the academic sector, 11% in government, 4% were employed in combined sectors (private, government and academic) and 4% were not practicing.

The mean knowledge score was 13 (±2.11) out of 18 and none of the respondents answered all questions correctly. The majority (80%) had ‘excellent’ knowledge scores and less than 2% had a ‘poor’ knowledge score. The mean vaccine knowledge score was 2.6 (±0.76) out of 4 with the majority (63%) having a score of 75%. More than half (60%) obtained a ‘good’ knowledge score while 40% obtained an ‘average’ score. There was no statistically significant difference between the knowledge categories and the mean age (*p* = 0.165); however, those who qualified in later years tended to have more participants in the ‘good’ knowledge category than those who graduated earlier (*p* = 0.029).

Over half (57%) of the respondents perceived COVID-19 as moderately dangerous while 38% believed it was not a serious public health issue. Almost all (95%) of the respondents believed that educating people about COVID-19 was important in preventing the spread of the virus. More than two-thirds (68%) preferred to avoid working with a COVID-19-positive patient, and almost 87% (283) received at least one vaccine against COVID-19.

Of the 13% (*n* = 43) who were not vaccinated, 93% (40) were worried about the possible adverse effects of the vaccines and felt there was not sufficient knowledge about the vaccines. Of those who received the vaccine, more than half (58%) experienced side effects such as pain at the injection site and fatigue.

With regard to willingness to administer the COVID-19 vaccines, 27% were willing to administer if allowed, and of these, 20% cited it would help to increase the public vaccination rate. Over 70% (*n* = 230) of respondents felt that the vaccines administered in SA were safe and that they were essential in the prevention of COVID-19 for OHCWs. Close to 90% felt that the vaccines should be fairly distributed to the population. The distribution of answers for the attitude questions is displayed in Table 1.

**TABLE 1:** Attitudes of oral healthcare workers regarding coronavirus disease 2019 and its vaccine.

Statements	Dentist	OH	DT	Specialist	DA	Total	
						*n*	%
**I prefer to avoid working with a patient who is suspect of having COVID-19 (*n* = 324)**							
Yes	133	38	14	16	20	221	68.0
No	62	12	0	13	16	103	32.0
**Total**	**195**	**50**	**14**	**29**	**36**	**324**	**100.0**
**Are you vaccinated against COVID-19? (*n* = 326)**							
Yes	174	38	14	26	31	283	87.0
No	21	12	0	3	5	41	13.0
**Total**	**195**	**50**	**14**	**29**	**36**	**326**	**100.0**
**If not, why? (*n* = 43)**							
Worried about the possible side effects of the vaccines	19	11	0	3	5	38	34.0
There isn’t sufficient knowledge about the vaccines	21	8	0	4	1	34	31.0
Vaccines developed and rolled out in record time	15	4	0	3	1	23	20.0
Mistrust in elected officials	10	5	0	0	2	17	15.0
**Total** (participants chose more than 1 option hence there were more than 43 responses)	**65**	**28**	**0**	**10**	**9**	**116**	**100.0**
**If you have been vaccinated against COVID-19, did you experience any side effects? (*n* = 318)**							
Yes	118	25	9	9	25	186	58.0
No	53	12	5	15	4	89	28.0
Not applicable	22	12	0	3	6	43	14.0
**Total**	**193**	**49**	**14**	**27**	**35**	**318**	**100.0**
If yes, what side effects did you experience?							
Pain at injection site	103	24	9	8	14	159	85.4
Fever	59	14	2	4	8	87	46.7
Headaches	62	14	5	4	15	100	53.8
Fatigue	62	17	6	5	13	103	55.4
Body aches	65	8	7	6	11	97	52.2
**Do you think your patients would be willing to receive a COVID-19 vaccine administered by you? (*n* = 320)**							
Yes	78	17	3	12	6	116	36.0
No	21	7	8	2	4	42	11.0
Maybe	95	25	3	13	26	162	53.0
**Total**	194	49	14	27	36	**320**	**100.0**
**The COVID-19 vaccine should be fairly distributed to everyone (*n* = 321)**							
Agree	176	39	14	25	31	285	89.0
Disagree	8	5	0	1	0	36	11.0
Undecided	10	5	0	2	5	22	10.0
**Total**	**194**	**49**	**14**	**28**	**36**	**321**	**100.0**

OH, oral hygienist; DT, dental therapist; Spec, specialist; DA, dental assistant; COVID-19, coronavirus disease 2019.

The majority of respondents (96%) reported to follow the recommended infection control policies and guidelines in their practices. The most common practices were to wear masks and making use of personal protective equipment (PPE) and waterless alcohol sanitiser. With regard to challenges in implementing infection control methods, only 17% of respondents stated that they experienced resistance from their patients and/or staff regarding infection control methods. More than half (60%) of participants believed that the role of the dentist in educating others about COVID-19 is very significant.

This distribution of answers for the practice questions is shown in Table 2.

**TABLE 2:** Types of practices regarding coronavirus disease 2019.

Questions/statements	Dentist	OH	DT	Specialist	DA	Total	
						*n*	%
**What infection control measures have you implemented at your workplace to reduce transmission of COVID-19? (*n* = 321)**							
Barriers and personal protective equipment (PPE)	167	43	12	26	21	269	82
Staff and patients to wear masks at all times up until time of consulting	173	46	12	27	28	288	88
UV light	21	4	3	4	1	33	10
Air purifiers	63	12	0	7	10	94	29
Redesign of waiting room to encourage social distancing	139	42	4	19	20	226	69
Completing a screening tool prior to their appointment	129	37	4	20	18	210	64
Temperature check on arrival	106	29	6	17	23	181	55
A total of 70% waterless alcohol sanitiser	161	40	10	25	27	265	81
All of the above	35	3	3	4	13	58	18
**What is the role of the dentist in teaching others about COVID-19? (*n* = 321)**							
Very significant	109	28	10	16	28	193	60
Moderately significant	58	11	2	7	5	83	26
Mildly significant	26	11	4	9	2	45	14

**Total**	**193**	**50**	**16**	**32**	**35**	**321**	**100**

OH, oral hygienist; DT, dental therapist; Spec, specialist; DA, dental assistant; COVID-19, coronavirus disease 2019.

## Discussion

The final sample size was 327, and this did not reach our calculated minimum sample size, which was 367. This could be because of the challenging times experienced by participants during the COVID-19 pandemic.

The mean age of the participants was 42 (standard deviation [SD] = ±12) years, which showed that many OHCWs were relatively experienced in SA. This could be because of the fact that almost a quarter of the respondents were employed in the academic sector, and these respondents tend to be older as academic institutions require specialists and experienced OHCWs. This was in contrast to a study performed in Nepal where the mean age of HCWs was 28 years (Limbu et al. 2020). Majority of the respondents received their latest qualification between 2005 and 2021, and this was expected as the majority of OHCWs within the age bracket would have qualified in the past 20 years.

The majority (60%) of respondents were dentists and among all respondents 55% had a postgraduate diploma and 32% a master’s degree, which was similar to a Jordanian study (Khader et al. 2020). In SA, the high number of respondents with postgraduate qualifications could be because of the fact that many feel the pressure to further their education in order to secure jobs, which could explain why over half of the respondents have a postgraduate diploma. In this study, 57% were employed in the private sector, 11% in the public sector and the remaining were either in academic, military or not practicing. This is consistent with the employment status of OHCWs in SA as reported in a previous study (Theodorea et al. 2021).

The majority (80%) of OHCWs had excellent knowledge scores regarding COVID-19. Those participants who had more years of experience had a ‘good’ knowledge score. This could be because the South African health system is embarking on awareness campaigns and disseminating relevant information among HCWs. This is comparable to a study carried out in Nepal, which reported that 82% of respondents demonstrated sufficient knowledge scores around COVID-19 (Limbu et al. 2020).

Overall, the vaccine knowledge was above average. This could also be as a result of the government awareness campaigns rolled out during the COVID-19 pandemic. The results were similar to an Indonesian study, which reported that all dental respondents were aware and wanted to learn about the vaccines for themselves and to educate their patients (Theodorea et al. 2021).

Over a third (38%) of the respondents considered it not to be a serious public health issue. This could be because when this study was being carried out, vaccines were readily available, which could have resulted in participants being less concerned about the seriousness and severity of the disease. These findings were similar to a study carried out in Jordan in which one-third believed COVID-19 was not a serious public health issue (Khader et al. 2020).

Just over 70% of the OHCWs felt that the vaccines administered in SA were safe, and 87% reported to have been vaccinated. This was considerably higher than a systematic review conducted among HCWs worldwide, which reported the vaccine acceptance rates to vary between 27% and 78% (Lowe et al. 2022). This suggests that among HCWs, the COVID-19 vaccination rates vary across geographic locations. The high rate reported in this study regarding the safety of vaccines could be because of fears regarding contracting and transmitting the virus as well as a fear of the financial implications of the virus, which prompted individuals to vaccinate. Also, there were many vaccination campaigns where vaccines were easily available.

Among the unvaccinated, contributing factors for vaccine refusal were concerns about possible side effects of the vaccines (34%), insufficient knowledge about the vaccines (31%) and vaccines developed and rolled out in record time (20%) and these findings were similar to a previous study (Chowdhury et al. 2022).

More than half (58%) of the respondents reported an adverse reaction post-vaccination. This included fatigue, followed by headaches, body aches and pain at injection site. This was similar to other studies, which also reported fever as a common side effect (Lowe et al. 2022).

The majority (96%) of the respondents followed the Centers for Disease Control and Prevention (CDC) guidelines for infection control practices. This could be because of the nature of the profession as OHCWs, as there is an increased risk for cross infection with SARS-CoV-2 between OHCWs and their patients (Turska-Szybka et al. 2021). This fact is reflected in the responses found in similar studies conducted in Saudi Arabia and Nepal where responses were 97% and 85%, respectively (Limbu et al. 2020; Srivastava et al. 2020).

## Conclusion

The majority of the respondents demonstrated a ‘good’ combined knowledge of COVID-19 and the vaccine and reported to be following the CDC guidelines for infection control. In general, a positive attitude towards COVID-19 was displayed; however, over a third of respondents in this study did not consider it a serious public health issue. The majority of respondents were vaccinated with more than half of them experiencing some form of side effect post vaccine.

### Recommendations

It is important that OHCWs are part of future vaccination processes. Efforts should be made to train dental therapists, oral hygienists and dentists to administer vaccines during emergency outbreaks as found during the height of the COVID-19 pandemic when there were not sufficient HCWs to administer the vaccines.

It is essential to evaluate the knowledge and attitudes of OHCWs around the virus and its recently discovered vaccines.

The vaccination hesitancy demonstrated among participants warrants further studies and evaluation into its efficacy and side effects as this could improve vaccination uptake with other similar outbreaks. Perhaps, developing tailored strategies to address concerns regarding the vaccine will be beneficial.

### Limitations

Although there were many responses around the attitudes and practices of OHCWs, a shortcoming was that these data can be challenging to measure. Even though the knowledge scores may be high, the attitudes and practices in this study may be affected by perception, which is immeasurable. The authors relied on good faith ensuring participants didn’t complete the questionnaire more than once.

The virus that causes COVID-19, SARS-CoV-2, is changing constantly; hence, new variants of the virus will continue to emerge. The knowledge around COVID-19 and the impact of the virus has changed quickly over time, and this could have resulted in some of the answers being incorrect at the time the data were collected.
