# Corrigendum: Knowledge, attitudes and practices of oral health workers regarding COVID-19 and its vaccine

**DOI:** 10.4102/hsag.v29i0.2816

**Published:** 2024-08-27

**Authors:** Zara Chothia, Ntombizodwa R. Nkambule, Ahmed Bhayat, Mpho Morule

**Affiliations:** 1 Department of Community Dentistry, Faculty of Health Sciences, University of Pretoria, Pretoria, South Africa

In the original article published, Chothia, Z., Nkambule, N.R., Bhayat, A. & Morule, M., 2024, ‘Knowledge and practices of South African oral’, *Health SA Gesondheid* 29(0), a2632. https://doi.org/10.4102/hsag.v29i0.2632, the title contained an incomplete title (factual error). The full title was not captured, which was unintentional. As a result of this, the title is corrected to read:

Knowledge, attitudes and practices of oral health workers regarding COVID-19 and its vaccine.

The authors apologise for this error. The correction does not change the study’s findings, its significance or overall interpretation of its results or the scientific conclusions of the article in any way.

